# The risk factors related to the severity of pain in patients with Chronic Prostatitis/Chronic Pelvic Pain Syndrome

**DOI:** 10.1186/s12894-020-00729-9

**Published:** 2020-10-07

**Authors:** Jing Chen, Haomin Zhang, Di Niu, Hu Li, Kun Wei, Li Zhang, Shuiping Yin, Longfei Liu, Xiansheng Zhang, Meng Zhang, Chaozhao Liang

**Affiliations:** 1 Department of Urology, The First Affiliated Hospital of Anhui Medical University, Hefei, People’s Republic of China; 2 The Institute of Urology, Anhui Medical University, Hefei, People’s Republic of China; 3 The Second Clinical College of Anhui Medical University, Hefei, Anhui People’s Republic of China; 4 Anhui Province Key Laboratory of Genitourinary Diseases, Anhui Medical University, Hefei, People’s Republic of China; 5 Department of Urology, b Reproductive Medicine Center, Xiangya Hospital, Central South University, Changsha, People’s Republic of China; 6 Institute of Urology of Shenzhen University, The Third Affiliated Hospital of Shenzhen University, Shenzhen Luohu Hospital Group, Shenzhen, People’s Republic of China

**Keywords:** Chronic prostatitis, Risk factors, Pain, Nomogram, Personalized treatment

## Abstract

**Background:**

Chronic Prostatitis/Chronic Pelvic Pain Syndrome (CP/CPPS) is a disease with diverse clinical manifestations, such as pelvic pain or perineal pain. Although recent studies found several risk factors related to the pain severity of CP/CPPS patients, results were inconsistent. Here, we aimed to identify novel risk factors that are closely related to the severity of pain in patients with CP/CPPS.

**Methods:**

We retrospectively collected the clinical records from patients with CP/CPPS from March 2019 to October 2019. The questionnaire was used to obtain related parameters, such as demographics, lifestyle, medical history, etc. To identify potential risk factors related to pain severity, we used the methods of univariate and multivariate logistic regression analyses. Further, to confirm the relationship between these confirmed risk factors and CP/CPPS, we randomly divided CP/CPPS patients into the training and the validation cohorts with a ratio of 7:3. According to the co-efficient result of each risk factor calculated by multivariate logistic regression analysis, a predicting model of pain severity was established. The receiver operating characteristic curve (ROC), discrimination plot, calibration plot, and decision curve analyses (DCA) were used to evaluate the clinical usage of the current model in both the training and validation cohorts.

**Results:**

A total of 272 eligible patients were enrolled. The univariate and multivariate logistic regression analysis found that age [odds ratio (OR): 2.828, 95% confidence intervals (CI): 1.239–6.648, *P* = 0.004], holding back urine (OR: 2.413, 95% CI: 1.213–4.915, *P* = 0.005), anxiety or irritability (OR: 3.511, 95% CI: 2.034–6.186, *P* < 0.001), contraception (OR: 2.136, 95% CI:1.161–3.014, *P* = 0.029), and smoking status (OR: 1.453, 95% CI: 1.313–5.127, *P* = 0.013) were the risk factors of pain severity. We then established a nomogram model, to test whether these factors could be used to predict the pain severity of CP/CPPS patients in turn. Finally, ROC, DCA, and calibration analyses proved the significance and stability of this nomogram, further confirmed that these factors were closely related to the pain severity of CP/CPPS patients.

**Conclusions:**

We identify age, holding back urine, anxiety or irritability, contraception, and smoking are risk factors closely related to the pain severity in patients with CP/CPPS. Our results provide novel inspirations for clinicians to design the personalized treatment plan for individual CP/CPPS patient who has suffered different encounters.

## Background

Prostatitis is a common urologic disease [1, 2], with studies reporting that during their lifetime, approximately 35–50% of men will suffer from prostatitis. The morbidity of prostatitis is higher in men who are not over 50-year-old [3]. According to previous work, we know that the prevalence rate of chronic prostatitis in Chinese males is approximately 8.4% [4]. Based on a proposal from the National Institutes of Health (NIH) [5], prostatitis is divided into four categories; of them, Category III, which is defined as chronic prostatitis or chronic pelvic pain syndrome (CP/CPPS), accounts for most cases of prostatitis [6]. CP/CPPS has a variety of clinical manifestations, such as pelvic pain or perineal pain, irritative or obstructive voiding symptoms, sexual dysfunction, or psychological disorders, and is without any evidence of urinary tract infection [7, 8]. Commonly, chronic pelvic pain occurs with pelvic floor tenderness, thus the patients will feel pain at the time of palpation [9].

Clinically, doctors always apply NIH-CPSI to judge the severity of chronic prostatitis. According to the subscores for pain, the pain levels are graded as mild (0 to 7), moderate (8 to 13), and severe (14 to 21). A recent study showed that the relationship between pain with the quality of life (QoL) in CP/CPPS patients was more important than urinary symptoms and the pain severity was more important than pain localization/type [10]. So, studying the risk factors for pain severity and clarifying the pain severity are helpful in improving the strategy of individualized phenotypically guided treatment. Although recent studies had found several risk factors related to the pain of CP/CPPS patients, results were inconsistent. Some studies had shown that a sedentary lifestyle, smoking, and stress were potential risk factors for the pain in patients with CP/CPPS [11]. But other studies showed that cigarette smoking was not related to the pain in patients with CP/CPPS [12]. Therefore, studying the risk factors for the pain severity in patients with CP/CPPS is particularly important for designing the personalized treatment plan.

In this study, we recorded the NIH-CPSI scores and other parameters from the outpatients with CP/CPPS to explore the risk factors related to the pain severity in patients with CP/CPPS and establish a predicting model of it.

## Methods

### Population selection

From March 2019 to October 2019, approximately 322 patients were diagnosis of CP/CPPS according to physical examination, microbiologic localization cultures, laboratory testing of stamey test, urodynamic studies and the UPOINTs classification [13] in the clinic of the First Affiliated Hospital of Anhui Medical University; we recorded the information from those patients. This study was approved by the Institutional Review Board of the First Affiliated Hospital of Anhui Medical University. Each patient had signed informed consent. The inclusion criteria for CP/CPPS patients were as follows: (1) symptoms lasting for at least 3 months; (2) discomfort or pain in the pelvic area or perineum that participants were excluded from the study if they had the urologic disease, such as acute prostatitis or bacterial prostatitis, urinary tract infection, urinary tuberculosis, bladder stone, interstitial cystitis, urethritis [14], and (3) the score of NIH-CPSI ≥ 4 [15]. The patients whose age was older than 50 years underwent a test of serum PSA based on the cut point of less than 4.0 ng/ml to exclude prostate cancer [16]. The patients who had benign prostatic hyperplasia (BPH) that the prostate ultrasound showed the prostate volume greater than 2*3*4 cm with the post-void residual urine volume of 50 ml were ruled out. Of all 322 recorded patients, 50 were excluded because of missing baseline values for continuous variables.

### Records of the variables

From the available records that we obtained from the CP/CPPS patients; according to some studies of the risk factors for CP/CPPS and the pain of CP/CPPS [11], the 11 variables were about basic information, lifestyle, and medical history were selected for further analysis. The sedentary lifestyle was defined as sitting or lying down when took part in an activity, such as reading, watching television, driving [17]. Holding back urine was defined as that waiting until the last second to go to the bathroom to pee [18]. In China, the main contraceptive method was the use of condoms, so in our study, the contraceptive method was set as the use of condoms. The questionnaire of the Self-Rating Anxiety Scale (SAS) was used to judge whether patients with CP/CPPS had the diagnosis of anxiety. When the scores of SAS was more than 50, the patients were diagnosed with anxiety [19]. 100 ml of beer per week differed significantly from 100 ml of liquor or spirits. To accurately assess the alcohol intake of patients, we uniformly defined the patient’s alcohol intake as grams of alcohol intake per week. According to the number of cigarettes daily in Paul’s study, we divided smokers into two groups: daily smoker of < 10 cigarettes and daily smoker of ≥ 10 cigarettes [20]. Skewed data were log-transformed or coded as categorical variables, and the detailed information was presented in Additional file 1: Table S1.

### Statistical analysis

**Fig. 1:**
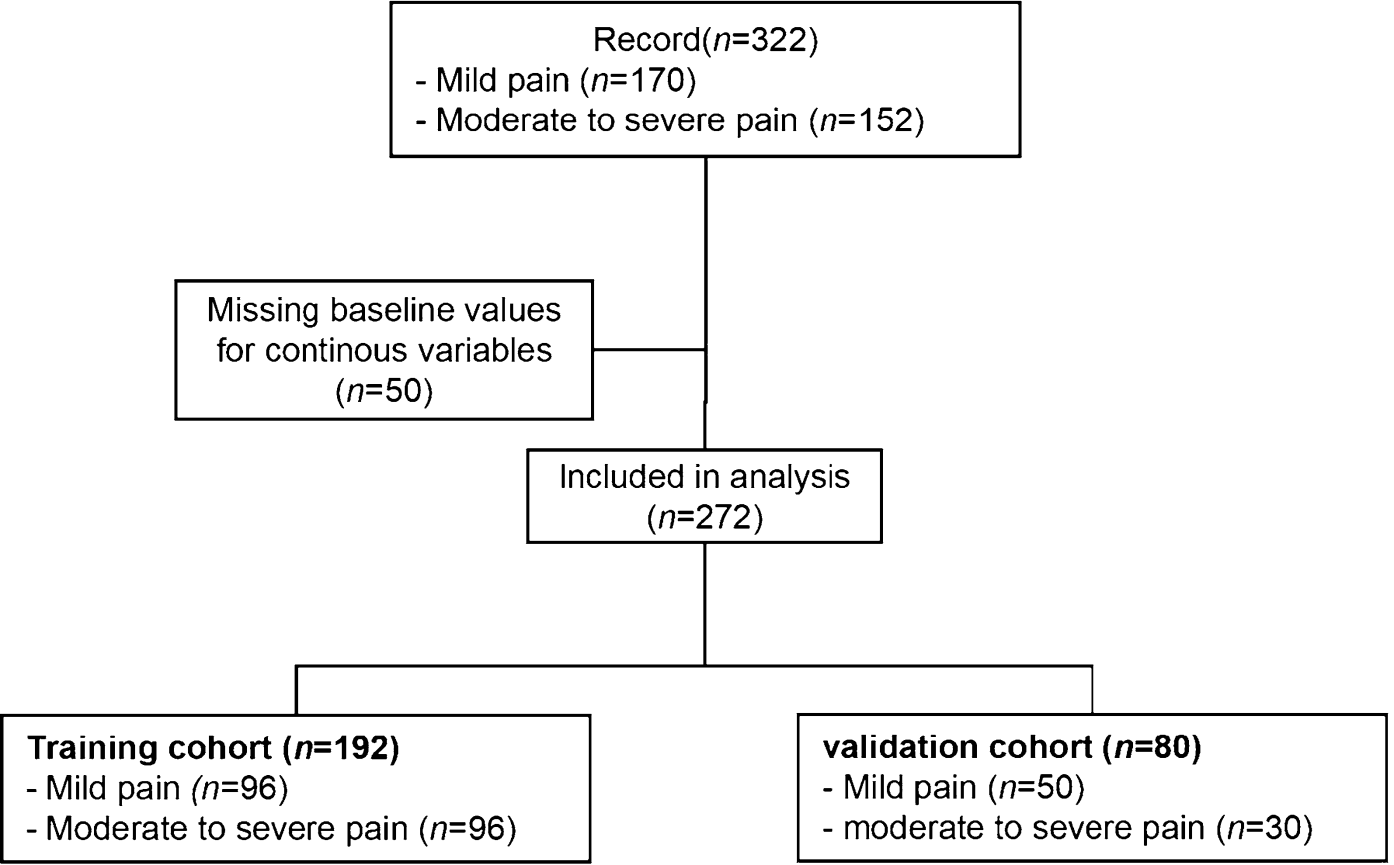
Prognostic analysis patient disposition

For the information of the patients, the normality was checked by the Shapiro–Wilk test and the demographic characteristics were compared by X^2^-test. When the p-value was lower than 0.05, the difference of risk factors between mild pain and moderately to severely pain groups was considered statistically significant. Univariate logistic regression analysis and multivariate logistic regression analysis were used to estimate those potential factors. The variables were considered as odds ratios (ORs), 95% confidence intervals (CIs), and *P*-values. To confirm the relationship between these confirmed risk factors and CP/CPPS, we randomly divided CP/CPPS patients into the training and the validation cohorts at a ratio of 7:3 (Fig. 1) to establish a predicting model of pain severity in patients with CP/CPPS. Based on these results of multivariate logistic regression analysis, a predicting model was created by using the “rms” package in R version 3.6.1. To assess the discrimination, calibration, and clinical usage of this predicting model, the methods of ROC, calibration plot, and DCA were executed. The validation cohort was used to confirm the significance and performance of this model.

## Results

### Risk factors for pain severity in patients with CP/CPPS

According to the NIH-CPSI’s subscores for pain, all patients were divided into two groups: CP/CPPS patients with mild pain (less than eight scores) and patients with moderate to severe pain (higher than a score of eight). The variables included age, BMI, white cells in the urine, sedentary, holding back urine, anxiety or irritability, sex life, contraception, past medical history, alcohol consumption, and smoking. The distributions of variables among CP/CPPS patients and the *P*-value of variables between the two groups were shown in Table 1. According to the univariate logistic regression analysis, age (*P* = 0.041), BMI (*P* = 0.007), holding back urine (*P* = 0.004), anxiety or irritability (*P* < 0.001), contraception (*P* = 0.012) and smoking (*P* < 0.001) were potential risk factors for the pain severity in patients with CP/CPPS. Multivariate logistic regression analysis was used to measure the variables to exclude the internal effects of the participants. We found that age [OR: 2.828, 95% CI: 1.239–6.648, *P* = 0.004], holding back urine (OR: 2.413, 95% CI: 1.213–4.915, *P* = 0.005), anxiety or irritability (OR: 3.511, 95% CI: 2.034–6.186, *P* < 0.001), contraception (OR: 2.136, 95% CI: 1.161—3.014, *P* = 0.029) and smoking (OR: 1.453, 95% CI: 1.313–5.127, *P* = 0.013) were the risk factors. The OR, 95% CI, and *P*-value of each variable were presented in Table 2.

**Table 1:** Comparison of risk factors in pain severity in patients of CP/CPPS

Characteristic	Mildly pain (n = 146)	Moderately to severely pain (n = 126)	*P* value
Age, years			
≤ 30 years	60 (41.10)	43 (34.13)	0.046*
30 to ≤ 40 years	52 (35.61)	36 (28.57)	
40 to ≤ 50 years	22 (15.07)	33 (26.19)	
> 50 years	12 (8.22)	14 (11.11)	
BMI (kg/m^2^)			
< 18.5 kg/m^2^	10 (6.85)	5 (3.97)	0.007**
18.5 to < 24 kg/ m^2^	84 (57.53)	50 (39.68)	
24 to < 27 kg/ m^2^	41 (28.08)	53 (42.06)	
≥ 27 kg/ m^2^	11 (7.54)	18 (14.29)	
White cell in urine, *n* (%)			
No	135 (92.46)	114 (90.48)	0.385
Yes	11 (7.54)	12 (9.52)	
Sedentary, *n* (%)			
No	77 (52.74)	54 (42.86)	0.066
Yes	69 (47.26)	72 (57.14)	
Holding back urine, *n* (%)			
No	123 (84.25)	88 (69.84)	0.004**
Yes	23 (15.75)	38 (30.16)	
Anxiety or irritability, *n* (%)			
No	88 (60.27)	43 (34.13)	< 0.001***
Yes	58 (39.73)	83 (65.87)	
Sex life, *n* (%)			
No	17 (11.64)	9 (7.12)	0.146
Yes	129 (88.36)	117 (92.88)	
Contraception, *n* (%)			
No	94 (64.38)	68 (53.97)	0.012*
Yes	52 (35.62)	58 (46.03)	
Past medical history, *n* (%)			
No	114 (78.08)	90 (71.43)	0.333
Urologic diseases	28 (19.18)	29 (23.02)	
Others	4 (2.74)	7 (5.55)	
Alcohol consumption, *n* (%)			
No	94 (64.38)	90 (71.43)	> 0.05
≤ 100 g/w	35 (23.97)	27 (21.43)	
> 100 g/w	17 (11.65)	9 (7.14)	
Smoking, *n* (%)			
No	110 (75.34)	8 (6.35)	< 0.001***
≤ 10 cigarettes/d	26 (17.81)	28 (22.22)	
> 10 cigarettes/d	10 (6.85)	90 (71.43)	

*BMI* Body Mass Index (obtained as weight divided by height squared)

**Table 2:** Multivariate logistic regression analysis found out the factors related to pain severity prediction

Parameters	OR	95% CI	*P* value
Age	2.828	1.239–6.648	0.004**
Holding back urine	2.413	1.213–4.915	0.005**
Anxiety or irritability	3.511	2.034–6.186	< 0.001***
Contraception	2.136	1.161–3.014	0.029*
Smoking	1.453	1.313–5.127	0.013*

*CI* confidential interval, *OR* odd ratio

*P < 0.05, **P < 0.01 and ***P < 0.001

### Establishment of the predictive nomogram

**Fig. 2:**
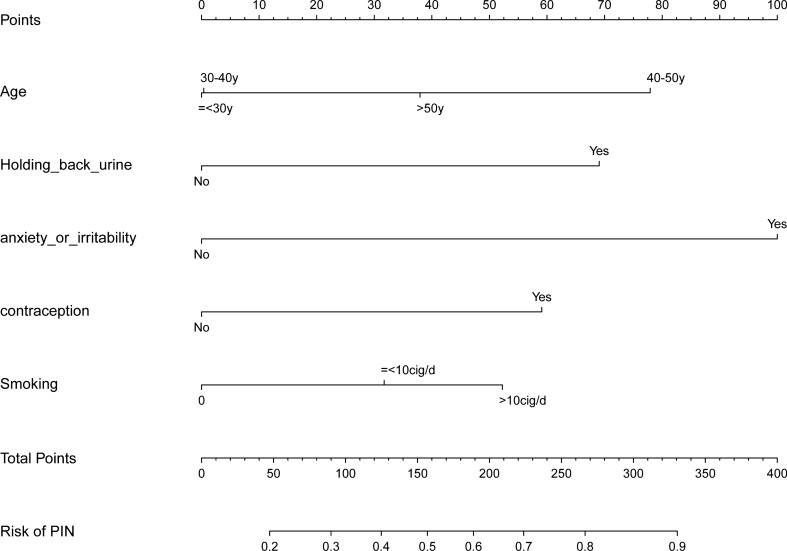
Novel nomogram of predicting the risk of the severity of pain of CP/CPPS patients. The severity of the pain of CP/CPPS patients nomogram was developed in the cohort, with the use of age, holding back urine, anxiety or irritability, contraception, and smoking

To confirm the relationship between these confirmed risk factors and CP/CPPS, a predicting model of pain severity in patients with CP/CPPS was established. The distributions of variables among CP/CPPS patients between mild pain and moderately to severely pain groups in the training and the validation cohorts were shown in Table 3. Based on the six variables derived from multivariate logistic regression analysis, we created a nomogram that could be used to predict the pain severity in patients with CP/CPPS. As shown in Fig. 2, the top bar indicated the scale for estimating the risk score of each variable, while the bottom bar corresponds to the pain severity in patients with CP/CPPS.

**Table 3:** Baseline patient and disease characteristics of the training and validation cohorts

Characteristic	Training cohort (n = 192)		Validation cohort (n = 80)	
	Mildly pain (n = 96)	Moderately to severely Pain (n = 96)	Mildly pain (n = 50)	Moderately to severely pain (n = 30)
Age, years				
≤ 30 years	37 (38.54)	31 (32.29)	23 (46.00)	12 (40.00)
30 to ≤ 40 years	36 (37.50)	29 (30.21)	16 (32.00)	7 (23.33)
40 to ≤ 50 years	17 (17.71)	27 (28.125)	5 (10.00)	6 (20.00)
> 50 years	6 (6.25)	9 (9.375)	6 (12.00)	5 (16.67)
Holding back urine, *n* (%)				
No	80 (83.33)	62 (64.58)	43 (86.00)	26 (86.67)
Yes	16 (16.67)	34 (35.42)	7 (14.00)	4 (13.33)
Anxiety or irritability, *n* (%)				
No	57 (59.375)	30 (31.25)	31 (62.00)	13 (43.33)
Yes	39 (40.625)	66 (68.75)	19 (38.00)	17 (56.67)
Contraception, *n* (%)				
No	62 (64.58)	50 (52.08)	32 (64.00)	13 (43.33)
Yes	34 (35.42)	46 (47.92)	18 (36.00)	17 (56.67)
Smoking, *n* (%)				
No	70 (72.92)	6 (6.25)	40 (80.00)	2 (6.67)
≤ 10 cigarettes/d	19 (19.79)	19 (19.79)	7 (14.00)	9 (30.00)
> 10 cigarettes/d	7 (7.29)	71 (73.96)	3 (6.00)	19 (63.33)

*BMI* Body Mass Index (obtained as weight divided by height squared)

### Apparent performance of the predictive nomogram in the cohort

**Fig. 3:**
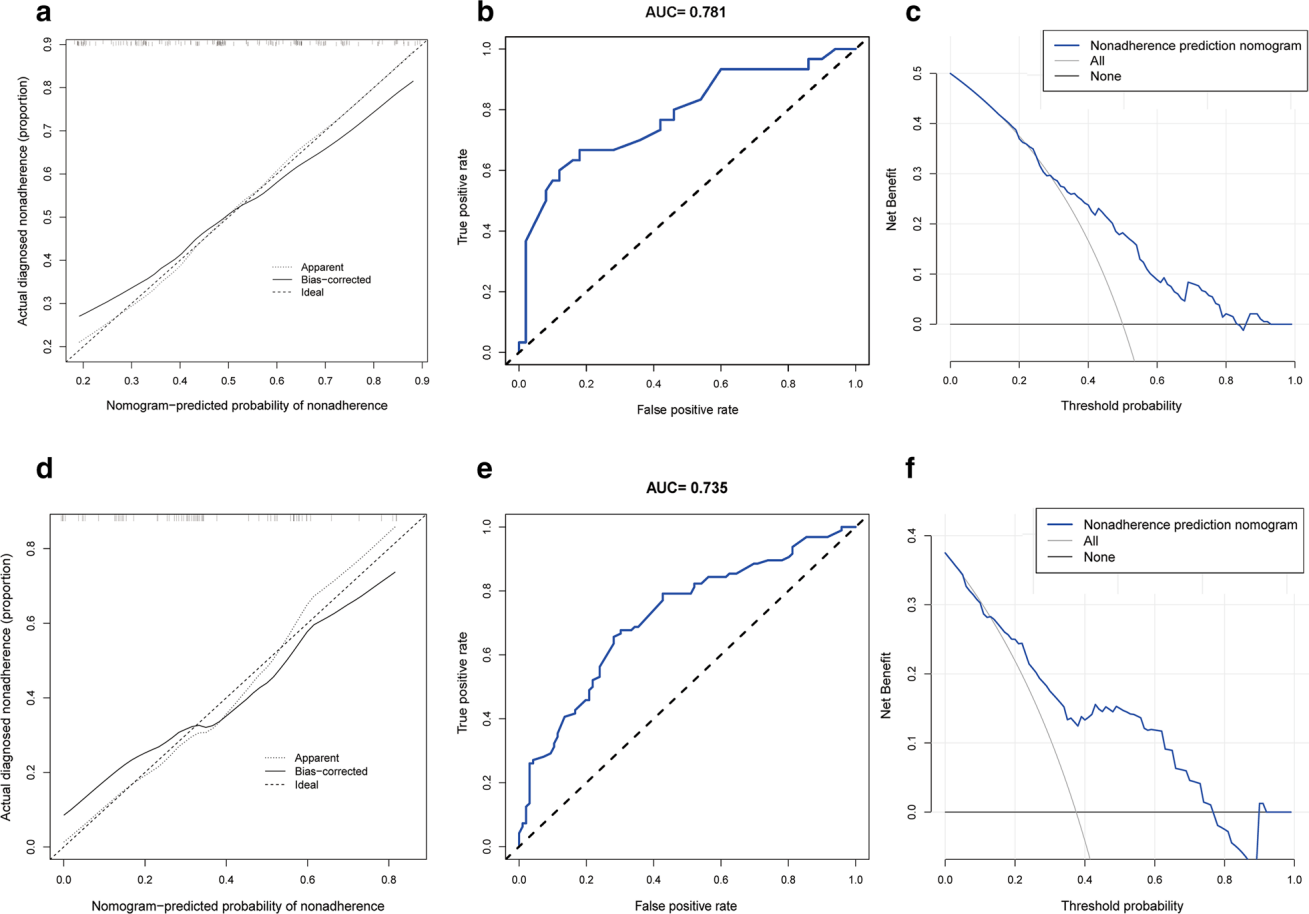
Apparent performance of the predictive nomogram in the cohort. Calibration curves of the pain severity nomogram prediction in the training cohort (**a**) and the validation cohort (**d**): The x-axis represents the predicted pain severity of CP/CPPS patients. The y-axis represents the actual pain severity of CP/CPPS patients. Receiver operating characteristic (ROC) curves of the nomogram in the training cohort (**b**) and the validation cohort (**e**): The ROC curve is displayed in solid line, and the reference is displayed in dotted line. The ROCs of the predictive nomogram in the training and validation cohorts, with the AUC of 0.781 and 0.735, respectively. Decision curve analysis (DCA) of the nomogram in the training cohort (**c**) and the validation cohort (**f**): The y-axis measures the net benefit. The blue solid line represents the pain severity predictive nomogram. The thin solid line represents the hypothesis that all patients are mild pain. The thin thick solid line represents the assumption that patients are moderate to severe pain. The DCA showed that if the threshold probability of a patient and a doctor are > 25% and < 83% in training cohort (**c**) and > 16% and < 78% in the validation cohort (**f**), respectively. Using this predictive nomogram to predict the pain severity of CP/CPPS patients adds more benefit than the intervention-all-patients scheme or the intervention-none scheme

To evaluate the calibration and discrimination of the nomogram, the calibration and ROC curves were applied. The calibration curve demonstrated a good agreement in the training cohort (Fig. 3a) and the ROC curve confirmed the predictive value of the nomogram, with the AUC value of 0.781 (Fig. 3b). Meanwhile, the DCA analysis was used to evaluate the clinical utility of this nomogram (Fig. 3c). We then used the validation cohort to verify the calibration, discrimination, and clinical utility of the nomogram. The calibration plot (Fig. 3d), AUC value (Fig. 3e), and DCA analysis (Fig. 3f) derived from the validation cohort showed similar results as the training cohort. These results reflected that the nomogram can precisely and steadily judge pain severity in patients with CP/CPPS.

## Discussion

Prostatitis is a common outpatient disease in urology. Recent research has shown that the prevalence rates of prostatitis in Europe and the USA are 10% to 14% [21]. In the USA, this health problem motivates 8% of urology consultations [1]. Among all types of prostatitis, CP/CPPS accounts for most cases. As shown in previous studies, men of all ages and ethnic origins can suffer from CP/CPPS, but the morbidity of the disease is more common in men who are younger than 50 years old [1]. Although the clinical presentations are diverse, the main clinical features of prostatitis are pelvic pain and lower urinary tract symptoms [22]. Thus, CP/CPPS is defined as pelvic pain that has presented for at least three months and for which no apparent cause has been found [23].

From recent research, we found that engagement in sedentary work and alcohol consumption had a negative influence while marriage had a positive impact on the prognosis of CP/CPPS [24]. Therefore, the questionnaire we designed included age, BMI, white cells in the urine, NIH-CPSI scores, sedentary, urinary retention, anxiety or irritability, sex life, contraception, past medical history, alcohol consumption, and smoking. Currently, nomograms are prognostic methods that can increase accuracy and make prognoses easier to understand, resulting in better clinical decision making; they are widely used in oncology and medicine [25]. Therefore, in our study, we used multivariate logistic regression analysis to figure out the risk factors for the pain severity in patients with CP/CPPS and used a nomogram device to predict pain severity in CP/CPPS patients. Through using logistic regression analysis to measure the variables, we found that age, urinary retention, anxiety or irritability, contraception, and smoking were related to the pain severity in patients with CP/CPPS and enrolled those variables in the predictive model. Incorporating these five variables into the nomogram allowed the prediction of pain severity in CP/CPPS patients and resulted in the construction of an accurate prediction model of pain severity in CP/CPPS patients. The validation cohort demonstrated good discrimination and calibration power.

For many diseases, age is a potential risk factor. Previous research showed that CP/CPPS was more prevalent in older people [26, 27]. Other research showed that younger age had been associated with more CP/CPPS symptoms [28] and worse QoL [29]. In our study, we found that age is a risk factor for pain severity in CP/CPPS patients, but the age ranges from 40 to 50 years had a higher risk for pain severity in CP/CPPS patients. In some opinions, sedentary and urinary retention could not cause CP/CPPS, but these variables could intensify pain severity among CP/CPPS due to the distention of the venous plexus of the prostate peripheral zone or chronic congestion of the pelvic cavity when in a sitting position [30]. In our study, we found that urinary retention had a significant correlation with the pain severity in CP/CPPS patients. In previous studies, alcohol consumption was related to unchanged or worse symptoms in CP/CPPS patients [24]. In our research, through the logistic regression analysis, we found that alcohol consumption was not connected with the pain severity in CP/CPPS patients.

Some researches showed that the frequency of sexual activity, especially the excessive number of sexual intercourse, was related to CP/CPPS [31]. So, we investigate the influence of sexual activity and contraception. In our study, we did not find the relationship between sexual activity with the pain severity in CP/CPPS patients. But we found that contraception was significantly related to the pain severity in CP/CPPS patients. According to previous research, condoms could delay ejaculation resulting in sex lasting longer [32]. In the process of a sexual impulse in humans, the pelvic congestion will regress in about 15–30 min after an orgasm or may last longer without an orgasm [30]. Prolonged sexual activity could increase pelvic congestion. So, one of the possible reasons that condoms could intensify pain severity among CP/CPPS may be due to increased pelvic congestion. Smoking tended to enhance pain sensitivity. However, whether smoking affects CP/CPPS is still controversial. In Chen’s study, they found that smoking is a harmful factor for CP/CPPS [11], but another study found that smoking resulted in a better symptom relief rate [24]. In our research, we found that smoking is a risk factor for pain severity in CP/CPPS patients.

The correlation between stress and pain severity in CP/CPPS patients has been rarely reported. One study showed that people who are under stress at home or work are 1.5-fold more likely to suffer from CP/CPPS than unstressed people [30]. Recently researches showed that biopsychosocial stress had a significant association with chronic pelvic pain in men [33]. In our study, we found that stress is a risk factor for pain severity in CP/CPPS patients. However, whether stress causes or results from the pain in CP/CPPS patients was difficult to decide in the present study.

A 2009 revision of the NIH-CPSI called the GU problem index (GUPI) is now the recommended index as it includes questions on pain with bladder filling and bladder emptying [34]. The questions on pain with bladder filling and bladder emptying did not include in the questionnaire we used according to the previous vision of NIH-CPSI. Then, we will add these two questions in our follow-up research.

## Conclusion

In summary, age, holding back urine, anxiety or irritability, contraception and smoking were related to the pain severity in patients with CP/CPPS, and the establishment of the nomogram model could accurately assess the pain severity in patients with CP/CPPS to provide novel inspirations for clinicians to design the personalized treatment plan for each CP/CPPS patient who has suffered different encounters.

## Supplementary information

**Additional file 1:** **Table S1.** Eleven variables were selected for analysis and variables tested.

## Data Availability

The datasets used and/ or analyzed during the current study available from the corresponding author on reasonable request.

## Abbreviations

CP/CPPS: Chronic Prostatitis/Chronic Pelvic Pain Syndrome
NIH: The National Institutes of Health
BPH: Benign prostatic hyperplasia
SAS: Self-Rating Anxiety Scale
CI: Confidence interval
ROC: Receiver operating characteristic curve
AUC: Area under the curve
DCA: Decision curve analyses
OR: Odds ratio
