# Supplementary material for: The risk factors related to the severity of pain in patients with Chronic Prostatitis/Chronic Pelvic Pain Syndrome
Source: BMC Urol. 2020 Oct 7;20:154. doi: 10.1186/s12894-020-00729-9 (PMC7542966; doi:10.1186/s12894-020-00729-9)
Supplement: Supplementary file 1 — Additional file 1: Table S1. Eleven variables were selected for analysis and variables tested. [file 12894_2020_729_MOESM1_ESM.docx]

**Table S1.** Fifteen variables selected for analysis and variables tested

| **Prognostic variable** | **Variable definition** |
| --- | --- |
| Age | Categories, age ≤ 30 years |
|  | Categories, 30 years < age ≤ 40 years |
|  | Categories, 40 years < age ≤ 50 years |
|  | Categorica, age > 50 years |
| BMI | Categories, BMI < 18.5 kg/m^2^ |
|  | Categories, BMI 18.5 to < 24 kg/m^2^ |
|  | Categories, BMI 24 to < 27 kg/m^2^ |
|  | Categories, BMI ≥ 27 kg/m^2^ |
| White cell in urine | No vs. Yes |
| Sedentariness | No vs. Yes |
| Holding back urine | No vs. Yes |
| Anxiety or irritability | No vs. Yes |
| Sex life | No vs. Yes |
| Contraception | No vs. Yes |
| Past medical history | No past medical history |
|  | Urologic diseases |
|  | others |
| Drinking | Categories, No Drinking |
|  | Categories, ≤ 100 g/w |
|  | Categories, > 100 g/w |
| Smoking | Categories, No Smoking |
|  | Categories, Smoking ≤ 10 cigarettes/d |
|  | Categories, Smoking > 10 cigarettes/d |

BMI, Body Mass Index; EPS, expressed prostatic secretion; w, week
